# Greater hippocampal gray matter volume in subjective hyperosmia: a voxel-based morphometry study

**DOI:** 10.1038/s41598-020-75898-6

**Published:** 2020-11-02

**Authors:** Pengfei Han, Franz Paul Stiller-Stut, Alexander Fjaeldstad, Thomas Hummel

**Affiliations:** 1 Interdisciplinary Center Smell and Taste, Department of Otorhinolaryngology, TU Dresden, Dresden, Germany; 2 The Key Laboratory of Cognition and Personality, Ministry of Education, Chongqing, China; 3 Faculty of Psychology, Southwest University, Chongqing, China; 4 Flavour Institute, Aarhus University, Aarhus, Denmark; 5 Flavour Clinic, Department of Otorhinolaryngology, Holstebro, Denmark

**Keywords:** Magnetic resonance imaging, Olfactory cortex, Human behaviour

## Abstract

Subjective hyperosmia refers to a self-reported olfactory ability that is superior to a normal, intact sense of smell (normosmia), and is associated with olfactory emotional experience. The current study used voxel-based morphometry to investigate the gray matter volume (GMV) in people with self-rated hyperosmia (subjective hyperosmia, SH, N = 18) in comparison to people with self-rated normal olfaction (subjective normosmia, SN, N = 14). Participants’ olfactory function were assessed by the extensive olfactory test battery, the “Sniffin’ Sticks” test. Within the predicted brain regions (regions-of-interest analyses), the SH participants showed larger GMV of the left hippocampus as compared to SN participants (FWE corrected *p* < 0.05). Further, the whole-brain search indicated that SH had larger GMV of the bilateral hippocampus, the right hypothalamus, the left precuneus, and the left superior frontal gyrus as compared to the SN group. ROI analyses showed positive correlations between the left hippocampal GMV and odor threshold or discrimination scores across all participants. In addition, the whole-brain analysis suggested that the self-rated olfactory ability was positively associated with GMV in the cerebellum, superior frontal gyrus and the precentral gyrus among SH participants. In conclusion, the current results suggest that SH was associated with increased GMV in several brain regions that were previously shown to be involved in the processing of cognitive aspects of odors.

## Introduction

Olfactory perception exhibits large inter-individual variations. Hyperosmia refers to an olfactory ability that is superior to a normal, intact sense of smell (normosmia)^1^ which includes subjects who reach at least the 90th percentile of the standardized Sniffin’ Sticks test score of the group aged 21–30 years (41.5 points or higher)^2^. People with hyperosmia are identified from 11 to 70 years, and their frequency peaks (around 11%) in the twenties^2^. Overall, clinically relevant hyperosmia seems to be a rare condition, although it is sometimes observed for specific odorants^3,4^.

Multiple studies have investigated the consistency between self-ratings of olfactory ability and the scores derived from standardized olfactory tests. Several studies found that patients with olfactory dysfunction could report their ability to smell with a relatively high accuracy^5–7^, although one study with large patient sample size (n = 1227) found nearly one-third patients with complete smell loss (anosmia) judged their sense of smell as at least “average”^8^. Such correlation between the olfactory functions derived from self-ratings and standardized psychophysical measurements becomes more ambiguous for normosmic people, with several studies reporting negative results^5,7–10^. Knaapila et al.^11^ found that in healthy participants, the self-rated olfactory acuity is associated with odor-evoked annoyance, which is related to negative valence (unpleasantness) of odors. Similarly, Knaapila and Tuorila^12^ noted that participants who regarded their sense of smell as exceptionally good reported more negative odor-related experiences than sex- and age-matched controls. In addition, people who regard novel objects’ odor as an important reason for liking or disliking them may assume this as an indicator for their particularly acute sense of smell^13^. Besides, it has been reported that the ability of creating olfactory mental images (e.g. the vividness of olfactory imagery) predicted self-evaluated olfactory function in both healthy participants and patients with smell loss^5^. Therefore, the self-reported superior olfactory performance seems to be associated with hypersensitivity towards the emotional and affective processing of olfactory stimuli.

In terms of the neural substrates of sensory hypersensitivity, one pervasive assumption in the literature is that individuals who are subjectively hypersensitive have greater neural responses to sensory stimuli^14^. Brain imaging studies using functional magnetic resonance imaging (fMRI) have suggested a link between sensory hypersensitivity and increased activity in limbic regions (amygdala, hippocampus). For example, groups known to report subjectively high sensory sensitivity such as autism^15,16^, have a greater BOLD response to auditory, visual or tactile stimuli. For the anatomical features, neurotypical variation in subjective hyper-sensitivity to sounds has been shown to be linked to increased gray matter volume of the auditory cortex but also a number of emotional processing related limbic regions, such as the amygdala and hippocampus^17^. Self-reported pain sensitivity rather than pain threshold was associated with increased gray matter volume in bilateral parahippocampal gyrus, extending to the hippocampus^18^. There were only few studies on the neural basis for olfactory hypersensitivity in literature. Professionals with long-time olfactory experiences showed increased gray matter volume in the insula and the entorhinal cortex as part of the olfactory system^19^. In a recent study, Wabnegger et al.^20^ showed that participants with hyperosmia (determined by Sniffin’ Sticks test with combined odor threshold, discrimination and identification [TDI] score over 41) had increased gray matter volume of the hippocampus and anterior insula. However, whether differences in self-evaluated olfactory function also show a neuroanatomical correlate, reflected by local differences in gray matter volume, is unknown.

In the present study, we used voxel-based morphometry (VBM) to investigate the gray matter volumes in a group with subjective hyperosmia (SH), and compared them to a group with subjective normosmia (SN), who have a self-reported normal sense of smell. Given that the emotional eloquent areas share function with olfactory processing, especially the limbic brain structures (hippocampus and amygdala), the orbitofrontal cortex (OFC) and the insula^21–24^, we focused on those brain regions and tempted to hypothesize that an increased gray matter volume in SH compared to SN participants.

## Methods and materials

### Participants recruitment

Participants underwent two steps of screening. First, participants were interviewed on phone about their sense of smell (provided with three choices: worse than normal, normal; better than normal). On the test day which took place at least one week after the telephone interview, participants were asked again to perform self-ratings for their olfactory performance on a 9-point scale (9 = extremely well, 8 = very well; 7 = much better; 6 = moderately better; 5 = normal, 4 = moderately worse; 3 = much worse; 2 = very bad; 1 = no perception). The SH participants were those who reported their sense of smell as “better than normal” over the phone interview, and also rated their sense of smell better than others (score higher than 5) on the test day. Similarly, SN participants were those who reported their sense of smell as “normal” during the phone interview and rated their sense of smell as normal (score = 5) on the test days.

In total 14 SH and 18 SN participants were included in the study. The participants’ demographical information are shown in Table 1. Participants were also screened to be free of major nasal pathology, which was ascertained by nasal endoscopy and a detailed, standardized interview. Their right-handedness were ascertained by a handedness questionnaire^25^. Other criteria for exclusion were pregnancy, neurological or psychiatric diseases, ferromagnetic implants (e.g., pacemakers and cochlear implants), previous head injury, or claustrophobia, smoking, and diseases or medication use known to be associated with olfactory dysfunction. The study was approved by the Ethics Committee at the TU Dresden (Protocol # EK262082010).

**Table 1:** Characteristics of the subjective normosmia (SN) and subjective hyperosmia (SH) groups.

	SN (N = 14)	SH (N = 18)	Comparison
Female/Male (n)	7/7	12/6	χ^2^ = 0.91, *p* = 0.47
Age (years)	36.3 ± 14.3	36.9 ± 13.1	t_30 =_ − 0.14, *p* = 0.89
Smell ability rating	5.0 ± 0.0	7.0 ± 0.9	F_(1,28)_ = 65.5, *p* < 0.001
Odor threshold	9.8 ± 3.2	9.6 ± 3.6	F_(1,28)_ = 0.13, *p* = 0.91
Odor discrimination	13.1 ± 2.1	13.4 ± 1.8	F_(1,28)_ = 0.07, *p* = 0.79
Odor identification	13.5 ± 1.3	14.2 ± 0.9	F_(1,28)_ = 2.55, *p* = 0.12
TDI score	36.4 ± 4.7	37.2 ± 4.5	F_(1,28)_ = 0.16, *p* = 0.69
IO-Association	18.2 ± 2.4	19.4 ± 2.6	F_(1,28)_ = 1.07, *p* = 0.31
IO-Application	16.9 ± 2.8	18.0 ± 2.7	F_(1,28)_ = 0.95, *p* = 0.34
IO-Consequence	17.5 ± 1.8	18.2 ± 2.7	F_(1,28)_ = 0.27, *p* = 0.61

Data are presented as means ± Standard Deviation.

TDI score indicates the composite score for odor threshold, discrimination, and identification.

IO, the importance of olfaction questionnaire.

### Olfactory test

Olfactory function was assessed using the “Sniffin’ Sticks” test (Burghart GmbH, Wedel, Germany)^26^, which is one of the most widely used standardized tests of olfactory performance, based on pen-like odor dispensing devices. The test assesses individual’s odor threshold (sensitivity), odor discrimination and odor identification performances. In the olfactory threshold test, odours were presented in 16 triplets of pens, two containing an odourless solvent and the other containing the target odor (2-Phenylethanol, rose-like smell) at a certain dilution (16 dilutions). The participants were asked to indicate the pen with the rose-like smell. Sensitivity was determined using a single-staircase, triple-forced choice procedure. Two successive correct identifications or one incorrect identification triggered a reversal of the staircase, i.e., the next higher or the next lower concentration step was presented, respectively. Seven reversals had to be obtained (including the starting point), and the sensitivity was defined as the mean of the last four staircase reversals. In the odour discrimination test, 16 triplets of pens, with two containing the same odor and the third a different one, were presented to the participants. Participants were asked to identify which of the three pens smelled differently. In the odor identification test, 16 common odors had to be identified from a list of four descriptors (multiple forced-choice procedure). For odor presentation, the experimenter removed the cap of the pens and held the stick for about 3 s at approximately 1–2 cm in front of both nostrils of the participants. The interval between two triplet presentations was approximately 20 s. Three olfactory tests were performed, always in the same order: odour detection threshold, odour discrimination, and odour identification. The composite odor threshold, discrimination and identification score (TDI) range from 1 to 48 points. The “Sniffin Sticks” test were conducted after participants had rated on the 9-point scales regarding their subjective evaluation of olfactory performances. This was to exclude the influence of psychophysical olfactory testing on the attention towards olfactory function among participants^9^. The Sniffin’ Sticks test took place in a quiet and well-ventilated room of the MRI facilities at TU Dresden immediately before MRI.

### Importance of Olfaction assessment

In addition to the Sniffin’ Sticks test, participants were asked about their individual significance of olfaction with the importance of olfaction (IOQ) questionnaire^27^. The 20-item IOQ consists of three subscales evaluating the emotions, memories and evaluations associated with odors (Association-scale), the degree of application of smells in daily life (Application-scale), and the importance of smells in decision making (Consequence-scale). Repectively, 6 items define 1 of 3 subscales (“application”, “association”, “consequence”). The 2 remaining items relate to the scale named “aggravation,”which captures the tendency to exaggerate the importance of olfaction. Participants answered each statement on a 4-point Likert scale (I totally agree, I mostly agree I mostly disagree, I totally disagree).The scale exhibits a good internal reliability (Cronbach’s α = 0.77) which capture the subjective awareness and attitude towards chemosensory stimuli, and is suitable for participants with normal or abnormal olfactory functions.

### Structural brain image acquisition

High-resolution T1 anatomical brain images were acquired on a 3 T Siemens Sonata scanner (model Trio, Siemens, Erlangen, Germany) with a 32-channel phased-array head coil. For each subject, the axial T1 weighted images (in total 192 slices) were acquired using a 3-dimensional magnetization-prepared rapid acquisition gradient echo (MPRAGE) sequence with the following parameter: voxel size: 0.73 × 0.73 × 1.0 mm; repetition time: 2180 ms; echo time: 3.93 ms; flip angle: 15°, field of view: 384 mm.

### Voxel-based morphometry

Voxel-based morphometry of T1 weighted images was performed using the CAT12 software (https://dbm.neuro.uni-jena.de/vbm/) implemented in SPM12 (Wellcome Centre of Imaging Neuroscience, Institute of Neurology, UCL, London, UK; https://www.fil.ion.ucl.ac.uk/spm) ran with MATLAB (version 2013a, The MathWorks, Natick, MA, USA). T1 images were first segmented into gray matter (GM), white matter (WM) and cerebrospinal fluid (CSF). The GM images were spatially normalized to a template in Montreal Neurological Institute (MNI) space using the high dimensional Diffeomorphic Anatomical Registration Through Exponentiated Lie Algebra (DARTEL)^28^. The quality control (homogeneity check) in CAT12 was applied to ensure high quality of the segmentation of GM from WM. Mean correlation of the GM segments (the unsmoothed segmentations that provide more anatomical details were used) across all participants were performed as a measure of image quality after pre-processing. This tool visualizes the correlation between the volumes using a boxplot and correlation matrices. Outliers (deviated greater than 2 standard deviations from the global mean) are indicated and were excluded from the sample before smoothing and statistical analyses. For both groups, no data was indicated as having a mean correlation more than 2 standard deviations (supplementary Figure S1). Finally, the normalized GM images were smoothed with a Gaussian kernel (full width at half maximum 8 mm). Automated data quality checks were performed as per the CAT12 toolbox. An absolute threshold masking value of 0.2 was applied to avoid possible edge effects between different tissue types^28^. The volumes of GM, WM and CSF of each participant were summed up to the total intracranial volume (TIV).

Gray matter volume (GMV) between the SH and SN groups was examined using the two-sample t-test model in SPM12, including sex, age, TDI score and TIV as covariates. The multiple regression approach implemented in SPM12 was used for correlation analyses between GMV and the following variables: Sniffin’ Sticks test scores, IOQ scores, and self-ratings of olfactory performances. Age, sex and TIV were included as covariates of interest in all the multiple regression models. Besides, for the correlation analysis between GMV and TDI score, self-ratings for olfactory performance was included as a covariate, in order to eliminate the group effect.

A voxel-intensity tests was conducted within the regions related to olfactory and emotional processing, including the amygdala, the orbitofrontal cortex (OFC), the insula and the hippocampus (Regions-of-interest [ROI] analyses). Anatomical masks of ROIs were defined from the WFU Pickatlas toolbox (ANSIR, Wake Forest University, Winston-Salem, NC, USA)^29^ based on the “automated anatomical labelling (AAL)” atlas^30^. The OFC mask was defined as follows: bilateral Frontal_Mid_Orb + bilateral Frontal_Inf_Orb + bilateral Front_Sup_Orb. Results were considered as significant with peak wise *p* < 0.05 family wise error corrected (pFWE_corrected_ < 0.05) after small volume correction across each individual ROI mask. All ROIs were tested simultaneously for the left and right hemispheres. We did not correct each individual ROI for multiple testing to minimize Type II errors.

In addition, the aforementioned analyses were also conducted on the whole-brain level. To control for multiple statistical testing, we maintained a cluster-level false-positive detection rate at *p* < 0.05 using a voxel-level threshold of *p* < 0.001 with a cluster extent (k) empirically determined by Monte Carlo simulations (n = 1000 iterations), by means of AlphaSim procedure^31^, implemented in the REST toolbox (https://www.restfmri.net/forum/REST_V1.7)^32^. With an initial uncorrected voxel-wise threshold of 0.001, the following cluster size was determined to achieve a cluster-level FWE corrected *p* < 0.05: k = 32 voxels (for comparison between groups), k = 23 voxels (for correlation between GMV and TDI score) and k = 24 voxels (for correlation between GMV and subjective olfactory ratings). Significant brain regions were labelled and reported with the AAL toolbox^30^.

### Statistical analysis

Statistical analyses were performed by means of SPSS (Version 24.0, SPSS Inc. Chicago, IL, USA) and GraphPad Prism (Version 6, GraphPad Software Inc., La Jolla, Ca, USA). The two-sample t-test was used to compare the age between groups. Chi-square test was used to compare the sex distributions. One-way ANOVA was used to compare the subjective ratings for olfactory function, the Sniffin’ Sticks test scores, and the importance of olfaction subscale scores, with age and sex included as covariates of interest. Data are presented as mean ± standard deviations (SD) and results were considered as significant with *p*-values below 0.05.

## Results

### Demographics and olfactory function

Olfactory performances measured by the Sniffin’ Sticks test showed no significant difference between the SH and SN groups. Among the SH group, seven participants rated their sense of smell as “moderately better (score 6)”, four participants as “much better (score 7)”, and seven participants as “very well (score 8)”. The importance of smell did not differ between SH and SN groups (Table 1).

### Group comparison of GMV

**Figure 1:**
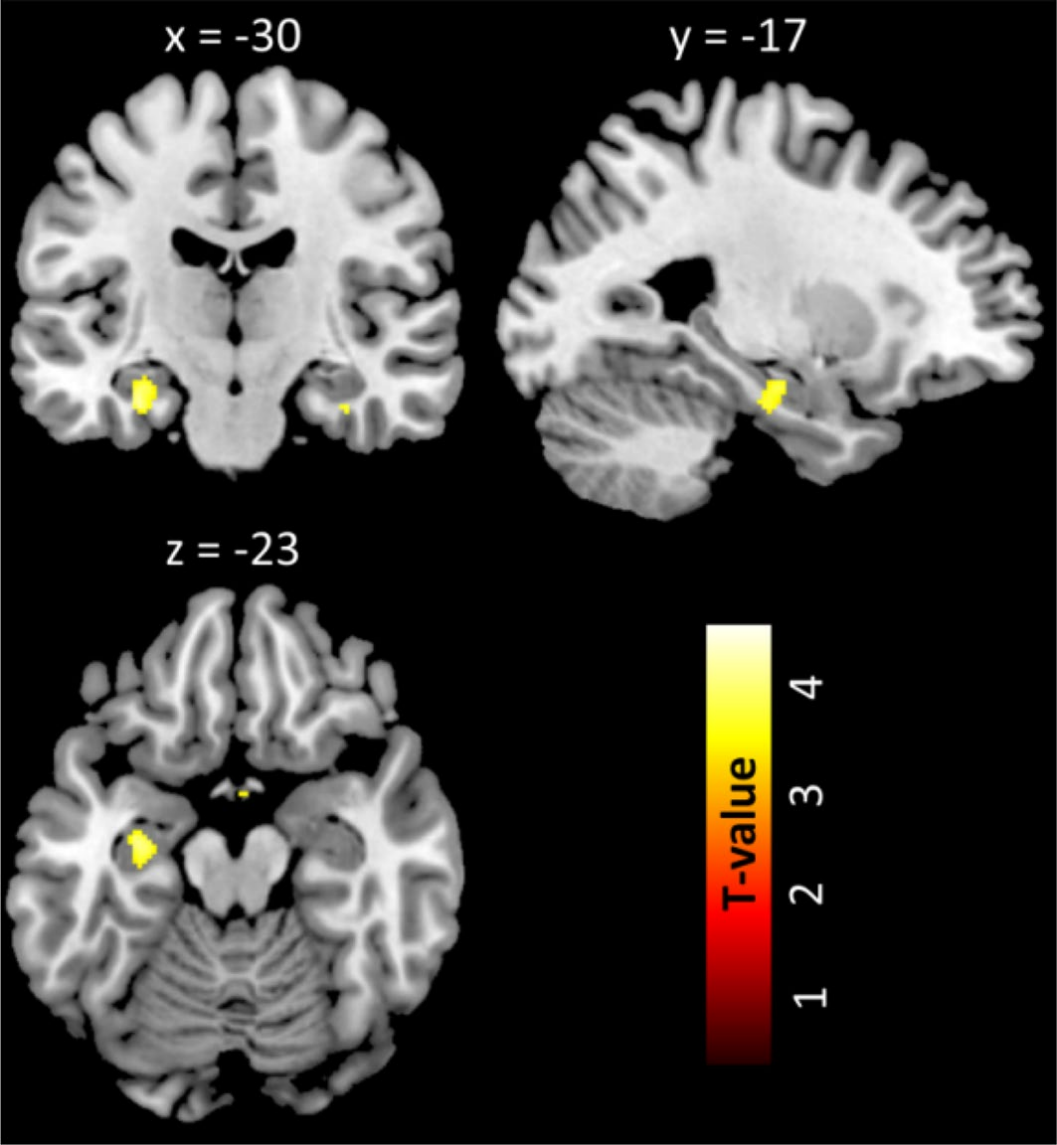
Larger gray matter volume of the left anterior hippocampus (pFWE_corrected_ < 0.05) in subjective hyperosmia as compared to subjective normosmia groups. Activation is visualized on a template provided by MRIcron (www.nitrc.org/projects/mricron). The T-map threshold was set at p_uncorrected_ ≤ 0.001 for visualization purpose.

**Figure 2:**
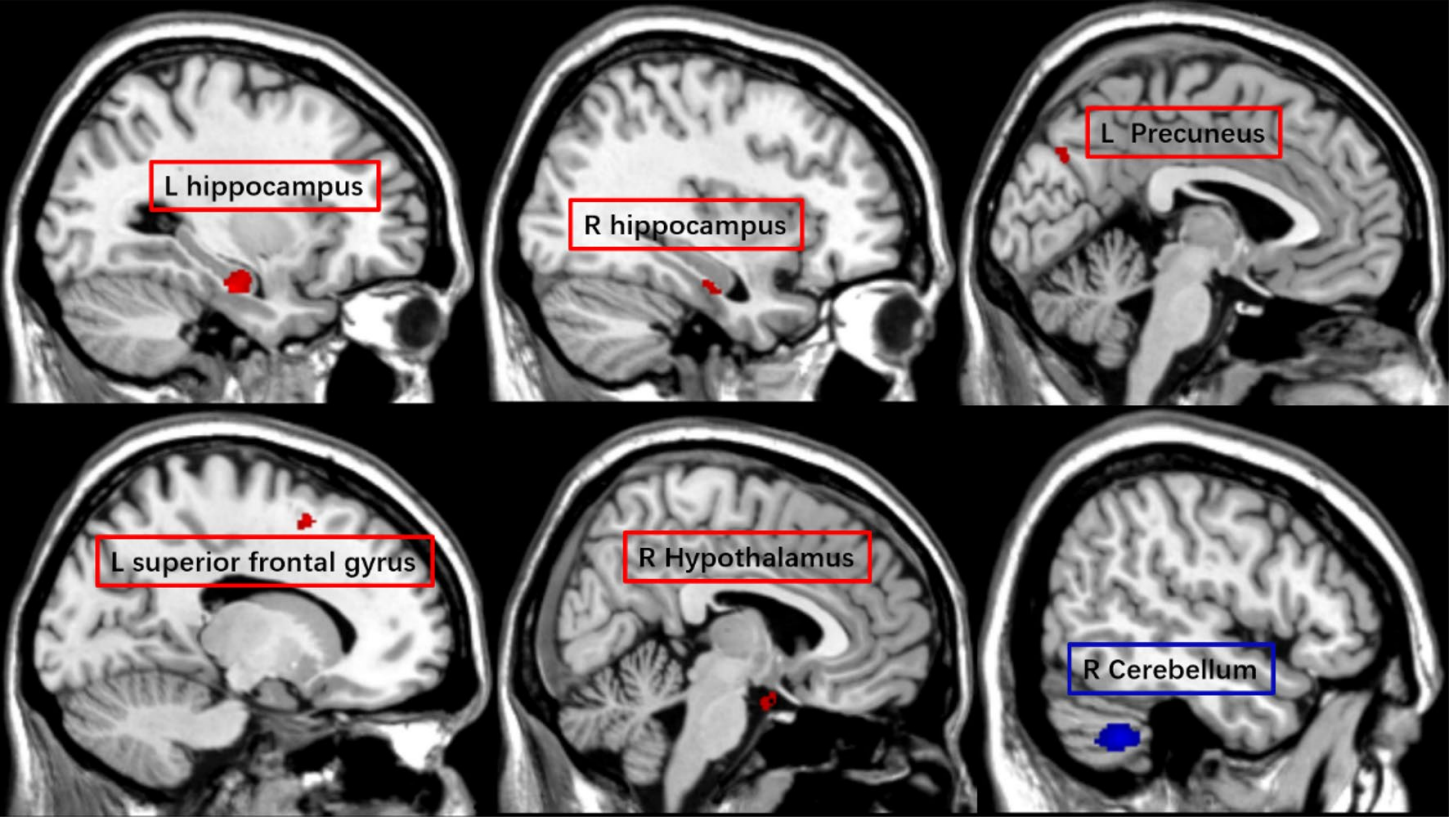
Whole-brain results (p_uncorrected_ < 0.001 and cluster size ≥ 32 voxels) of the gray matter volume (GMV) difference between subjective hyperosmia (SH) and subjective normosmia (SN) groups. Red clusters indicate larger GMV for SH > SN; Blue cluster indicates larger GMV for SN > SH. Activation of the significant cluster is visualized on a template provided by MRIcron (www.nitrc.org/projects/mricron).

The ROI analyses showed significantly enhanced GMV in the left hippocampus (cluster peak coordinates − 30 − 17 − 23; T = 4.95; pFWE_corrected_ = 0.015, k = 131 voxels, Fig. 1) in SH group relative to the SN group, no significant result was observed for the SH < SN contrast. The whole-brain analysis further revealed significantly larger GMV of the right hippocampus, right hypothalamus, left superior frontal gyrus and left precuneus among the SH group compared to the SN group (corrected *p* < 0.05; Table 2 and Fig. 2). For the reversed contrast (SN > SH) revealed a cluster in the right cerebellum (Table 2 and Fig. 2).

**Table 2:** Brain morphological (gray matter volume) differences between subjective hyperosmia (SH) and subjective normosmia (SN) groups.

	Cluster size (voxels)	Peak T value	MNI coordinate xyz (mm)			Brain region (AAL)
SH > SN	153	4.92	− 30	− 17	− 23	Hippocampus L*
	56	4.08	32	− 18	− 23	Hippocampus R
	59	4.62	6	− 2	− 24	Hypothalamus R
	38	4.42	− 15	11	54	Superior Frontal Gyrus L
	60	3.86	− 5	− 74	38	Precuneus L
SH < SN	502	5.96	57	− 45	− 42	Cerebellum R

Results presented were threshold at p_uncorrected_ < 0.001 and cluster size ≥ 32 voxels on a whole-brain level.

*significant with ROI analyses.

### Correlation between regional GMV and other variables

**Figure 3:**
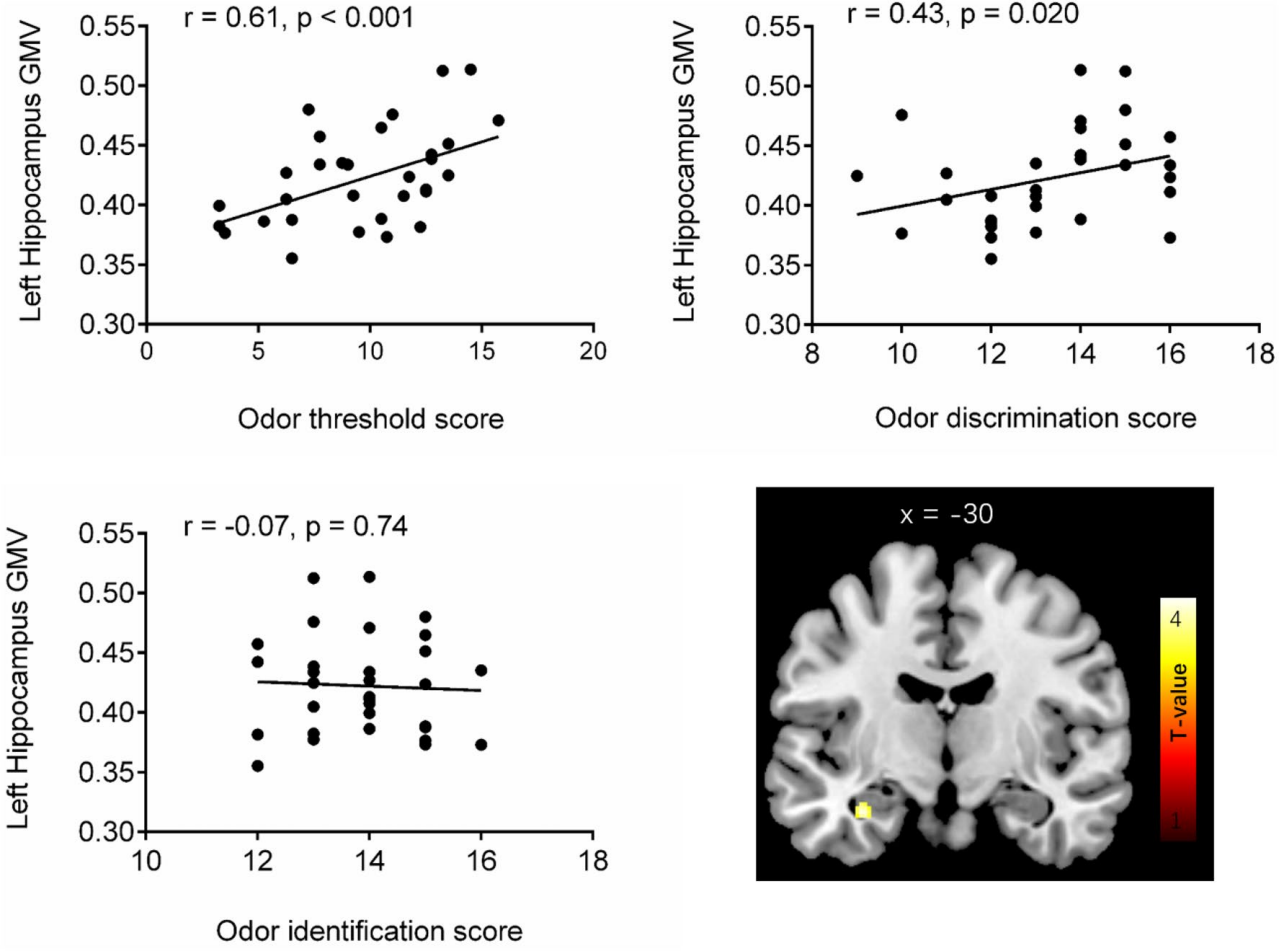
Correlations between the left hippocampal gray matter volume (cluster peak coordinates − 32 − 12 − 23; T = 4.57; pFWE_corrected_ = 0.034, k = 51 voxels) and the Sniffin’ Sticks odor threshold and discrimination score. Activation of significant cluster is visualized on a template provided by MRIcron (www.nitrc.org/projects/mricron). The T-map was threshold was set at p_uncorrected_ ≤ 0.001 for visualization purpose.

**Figure 4:**
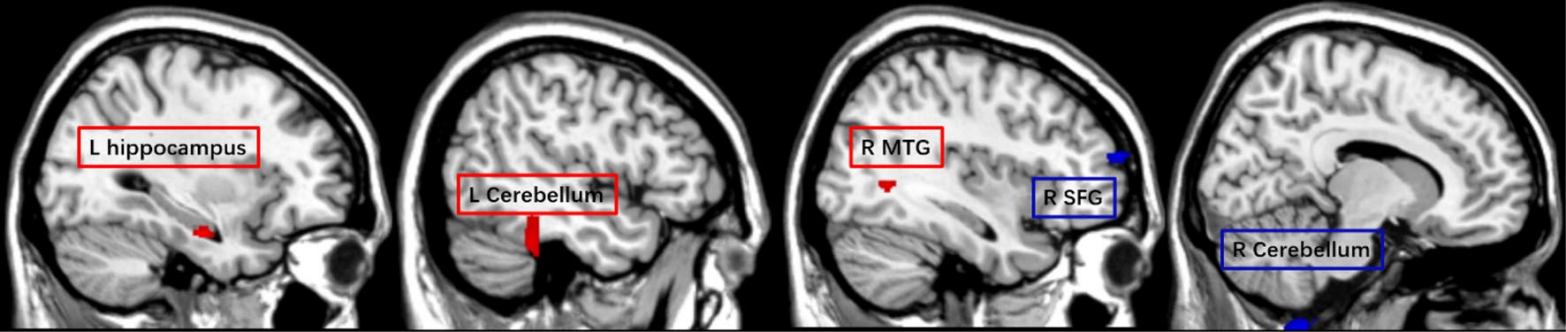
Whole-brain results (p_uncorrected_ < 0.001 and cluster size ≥ 24 voxels) of the gray matter volume showing correlation with Sniffin’ Sticks test scores among the whole study sample (N = 32). Brain regions with positively correlations are depicted in red, and with negative correlations are depicted in blue. Activation of the significant cluster is visualized on a template provided by MRIcron (www.nitrc.org/projects/mricron).

For all participants (N = 32), the ROI analyses found significant positive correlation between the GMV of the left hippocampus (peak coordinates − 32 − 12 − 23; T = 4.57; pFWE_corrected_ = 0.034, k = 51 voxels) and TDI scores. Extracted GMV from this significant cluster were found correlated to Sniffin’ Sticks score for odor threshold (r = 0.61, *p* < 0.001) and odor discrimination (r = 0.43, *p* = 0.020), but not for odor identification (r = − 0.07; *p* = 0.74) (Fig. 3). Whole-brain analyses showed positive correlation between the TDI score and the GMV of the left hippocampus the left cerebellum, and the right middle temporal gyrus (Table 3 and Fig. 4). Negative association between TDI score and GMV was observed for the right cerebellum and right superior frontal gyrus (Table 3 and Fig. 4).

**Table 3:** Correlation between TDI score and GMV across all participants (N = 32).

	Cluster size (voxels)	Peak T value	MNI coordinate xyz (mm)			Brain region (AAL)
Positive	69	4.57	− 32	− 12	− 23	Hippocampus L*
	186	4.44	− 50	− 39	− 30	Cerebellum L
	132	4.08	38	− 59	2	Middle temporal gyrus R
Negative	185	4.97	12	− 47	− 71	Cerebellum R
	86	4.79	39	63	17	Superior frontal gyrus R

Results presented were threshold at p_uncorrected_ < 0.001 and cluster size ≥ 24 voxels; *significant with ROI analyses.

**Figure 5:**
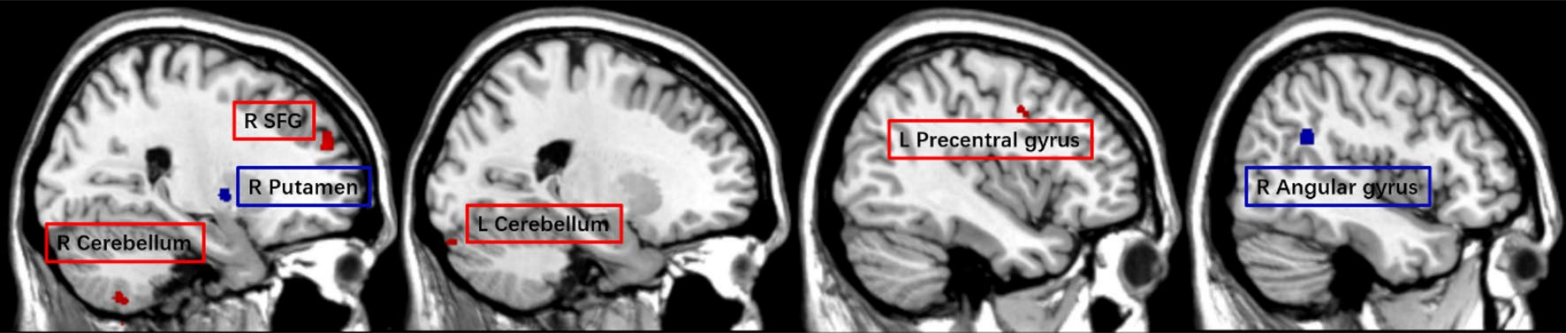
Whole-brain results (p_uncorrected_ < 0.001 and cluster size ≥ 24 voxels) of the gray matter volume showing correlation with subjective ratings of olfaction among the subjective hyperosmia group (N = 18). Brain regions with positively correlations are depicted in red, and with negative correlations are depicted in blue. Activation of the significant cluster is visualized on a template provided by MRIcron (www.nitrc.org/projects/mricron).

For the SH participants (N = 18) and with the whole-brain analysis, the self-ratings for olfactory performance was positively associated with the GMV in the bilateral cerebellum, the right superior frontal gyrus, and the left precentral gyrus; and negatively associated with GMV of the right putamen and the right angular gyrus (Table 4 and Fig. 5). No significant cluster was observed with ROI analyses.

**Table 4:** Correlation between self-ratings for olfactory performance and GMV among the subjective hyperosmia (SH) group (N = 18).

	Cluster size (voxels)	Peak T value	MNI coordinate xyz (mm)			Brain region (AAL)
Positive	208	5.68	29	− 53	− 68	Cerebellum R
	163	5.05	24	50	26	Superior frontal gyrus R
	24	4.45	− 24	− 92	− 24	Cerebellum L
	24	4.21	− 41	2	41	Precentral gyrus L
Negative	219	7.23	30	− 3	2	Putamen R
	175	5.19	44	− 48	26	Angular gyrus R

Results presented were threshold at p_uncorrected_ < 0.001 and cluster size ≥ 24 voxels.

## Discussion

The current study compared the regional gray matter volume (GMV) between subjective hyperosmia (SH) and subjective normosmia (SN) participants. Results revealed a larger left anterior hippocampal GMV in SH as compared to SN, although the “Sniffin’ Sticks” test scores were not different between the two groups. The hippocampus is a key region involved in a variety of olfactory processes, including odor discrimination^33^, odor familiarity processing^34^, episodic odor memory^35^ and odor-visual integration^35,36^. Following a coordinate-based segmentation for the hippocampus, the cluster showing increased GMV among SH participants from the current study was part of the anterior hippocampus (anterior to y = -21 mm in MNI space)^37^. There are substantial evidence showing that the subregions along the hippocampal anterior–posterior axis are associated with different functional specializations. The anterior part of the left hippocampal formation has been reported to specialize in emotional processing^37–39^, particularly the encoding of emotional memories^40–42^, as well as modality-independent memory retrieval^43^. Studies regarding the specific role of the anterior hippocampal subregions in olfactory process is limited. However, a coordinate search revealed that the anterior hippocampus is involved in cross-modal emotional processing of odor pleasantness^44^, as well as in olfactory-related emotional memory^45^, cross-modal recognition memory^35,46^.

It had been found that the self-rating of superior olfactory function correlated with higher degree of self-rated odor annoyance^11^, and a tendency to be influenced by odors in their liking (and not liking) for new stimuli than other participants^47^. Studies have shown the association between self-reported sensory sensitivity and structural change of the hippocampus. For example, self-reported pain sensitivity was positively associated with larger GMV in the hippocampus and parahippocampus, however no GMV changes was found in relation to the pressure pain threshold^18^. Neurotypical hyper-sensitivity to sounds, assessed with questionnaire, has been shown to be linked to increased GMV in the auditory cortex but also in limbic regions including the bilateral hippocampus^17^. Thus, a larger hippocampus may be associated with subjective sensory sensitivity in general. Historically, sensory sensitivity has been linked to a sensitive personality type that includes introversion and increased emotionality^48^. Hence, we may speculate that subjective sensory hypersensitivity is related to the ability to form the associations between sensory stimuli and emotional experiences, and the hippocampal GMV serve as a neural mediator in this association. However, the odor related annoyance or affective attitudes were not measured in our study, which limits further test on the functional role of the hippocampus. Besides, the cross-sectional design in those studies, makes it impossible to detect causal relationships. Perhaps, self-reported hyperosmic individuals are born with a predisposition for larger volumes of the hippocampus, leading them to be more prone to evaluate the emotional sensory stimuli. Inferring the functional meaning of local GMV alterations as detected by VBM is difficult although links between the GMV and task-related brain activation had been shown^49^. An intriguing question for future study is to investigate the hippocampal brain responses to emotional olfactory cues among SH people.

We also found a positive association between the GMV of the anterior hippocampus and odor sensitivity and discrimination scores. It is noted that the significant cluster was close to the one from the between-group comparison, suggesting the potential role of this part of the hippocampus in integrating olfactory perception, olfactory emotion and episodic olfactory memory retrieval. In an earlier study with 112 healthy participants, Smitka et al.^50^ found a significant correlation between the GMV of the right hippocampus and odor threshold. Recently, Wabnegger et al.^20^ showed that hyperosmic participants (“Sniffin’ Sticks” TDI score over 41) had larger GMV of the posterior subregion of the hippocampus (cluster peak coordinates posterior to y = − 21 mm in MNI space). Hence, both studies show positive associations between TDI score and GMV in the hippocampus. It was not clear from the Wabnegger’s paper which specific olfactory functions (e.g. odor threshold, discrimination and identification) was significantly correlated to the hippocampal GMV. It is possible that the anterior and posterior subregions of the hippocampus serve different roles in specific olfactory functions. The specific functions for the anterior–posterior hippocampal subregions could also explain the weak (although significant) correlation between olfactory functions and the whole right hippocampus GMV, and the null findings between olfaction and the left hippocampus GMV reported in the paper from Smitka et al.^50^.

Beyond the predicted regions of interest, the SH group also showed increased GMV in the left precuneus, left superior frontal gyrus and the right hypothalamus. Precuneus is involved in odor-evoked autobiographical memory, mental imagery or olfactory memory retrieval^51,52^. The hypothalamus, as a secondary olfactory area, receives projections from the primary olfactory cortex and appears to be involved in odor processing under emotional context^35,53^. However, there is lack of evidence supporting its role in subjective hyperosmia or general sensory sensitivity.

There are limitations to the current study. First, although the ROI analyses had controlled for multiple comparisons, it is necessary to validate the current research findings with a larger sample. Second, the variation in normalisation accuracy or smoothness during the VBM analyses is likely to result in statistical sensitivity varying over different brain regions, which is one major limitation for the VBM technique^54^. Thus, the absence of a statistically significant effect in any particular region does not prove that the region is unaffected. In addition, the null finding regarding the importance of olfaction between SH and SN groups may suggest the specificity of the questionnaire. Future study combining brain imaging techniques with psychometric assessment regarding the olfaction related affective responses (e.g. the affective impact of odor scale) or odor awareness (e.g. the odor awareness scale) should obtain deeper knowledge about the neurobehavioral basis of subjective hyperosmia.

In conclusion, the current study suggested that self-rated hyperosmia is associated with increased GMV in the anterior hippocampus. The GMV of this region also showed positive correlation to the odor threshold and odor discrimination scores. Future research should elaborate on the neural basis for emotional and perceptual olfactory processing.

## Ethical approval

All procedures performed in studies involving human participants were in accordance with the ethical standards of the institutional and/or national research committee and with the 1964 Helsinki declaration and its later amendments or comparable ethical standards. The study was approved by the Ethics Committee at the TU Dresden (Protocol # EK262082010).

## Informed consent

Informed consent was obtained from all individual participants included in the study.

## Supplementary information

Supplementary Information
