# Supplementary material for: Greater hippocampal gray matter volume in subjective hyperosmia: a voxel-based morphometry study
Source: Sci Rep. 2020 Nov 2;10:18869. doi: 10.1038/s41598-020-75898-6 (PMC7608672; doi:10.1038/s41598-020-75898-6)
Supplement: Supplementary file 1 — Supplementary Information [file 41598_2020_75898_MOESM1_ESM.doc]

Greater hippocampal gray matter volume in subjective hyperosmia: a voxel - based morphometry study

Pengfei Han^1,2,3*^, Franz Paul Stiller-Stut ^1^, Alexander Fjaeldstad ^4, 5^, Thomas Hummel^1^

^1^ Interdisciplinary Center Smell and Taste, Department of Otorhinolaryngology, TU Dresden, Dresden, Germany

^2^ Faculty of Psychology, Southwest University, Chongqing, China

^3^ Key Laboratory of Cognition and Personality (Southwest University), Ministry of Education, Chongqing, China

^4^ Flavour Institute, Aarhus University, Aarhus, Denmark

^5^ Flavour Clinic, Department of Otorhinolaryngology, Holstebro, Denmark

*Corresponding author; [pengfeihan@swu.edu.cn](mailto:pengfeihan@swu.edu.cn);

**Supplementary materials**


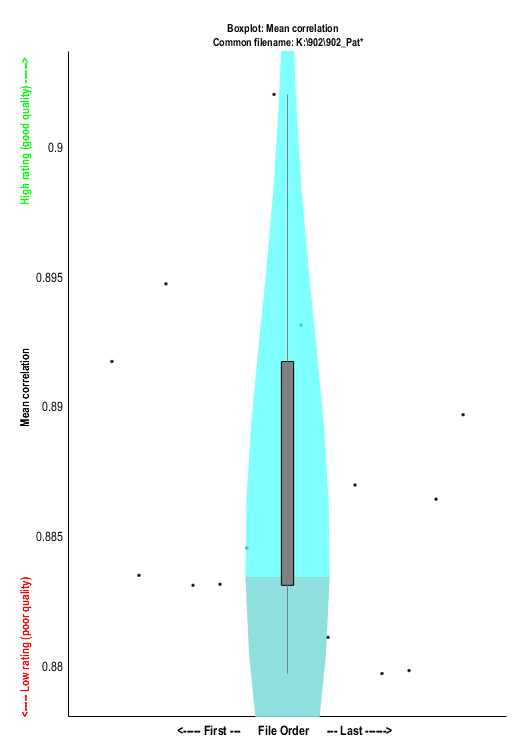

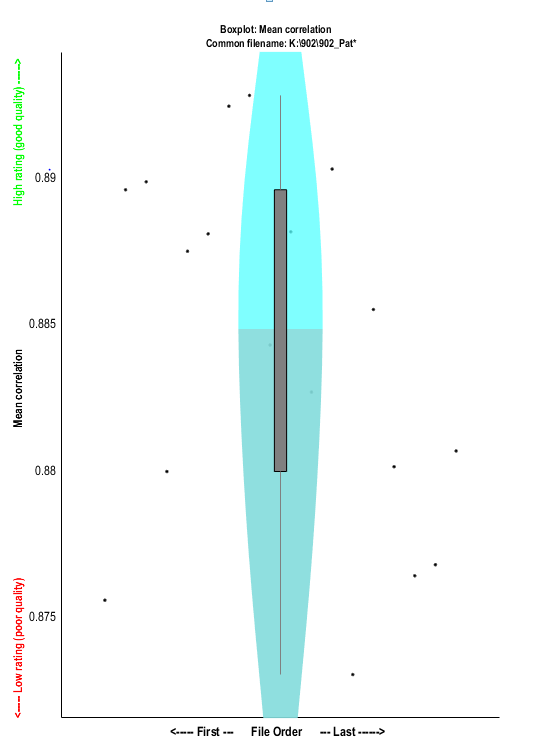


Figure S1 Mean correlation between gray matter segments of individual datasets in subjective normosmia (left) and in subjective hyperosmia (right) groups. For both groups, no data point was deviated more than 2 standard deviations of the mean, indicating good gray matter segmentation quality.
